# Hypoglycemic and Hepatoprotective Effects of Dried and Rice-Fried *Psidium guajava* Leaves in Diabetic Rats

**DOI:** 10.1155/2021/3346676

**Published:** 2021-12-02

**Authors:** Dan Li, Sen Yang, Hai-yan Ding, Hong-ping Chen, You-ping Liu, Yuan Hu

**Affiliations:** State Key Laboratory of Traditional Chinese Medicine Resources of Southwest China, and Key Laboratory of Chinese Medicine Standardization Ministry of Education, Chengdu University of Traditional Chinese Medicine, Chengdu 611137, China

## Abstract

*Psidium guajava* leaves (PGL) have been long used as an adjuvant therapy for diabetics. The present study evaluated the in vivo hypoglycemic and hepatoprotective effects of dried and the rice-fried PGL decoctions (PGLD and RPGLD). Our results indicated that both PGLD and RPGLD could significantly decrease the contents of fasting blood glucose (FBG) and haemoglobin A1c (HbA1c) in diabetic rats. Compared with the HFD/STZ (high-fat diet with streptozotocin) group, the PGLD and RPGLD-treated diabetic rats showed different degrees of recovery against the liver pathological changes. The upregulated expressions of glucokinase (GK), glucose transporter 2 (GLUT2), insulin growth factor-1 (IGF-1), insulin receptor substrate-1 (IRS-1), and insulin receptor substrate-2 (IRS-2) in PGLD and RPGLD-treated groups were observed. In general, RPGLD exhibited a much better antidiabetic effect than PGLD, which was further verified by the comprehensive evaluation with the TOPSIS method. Besides, HPLC (high-performance liquid chromatography) and UPLC-MS/MS (ultra-performance liquid chromatography-tandem mass spectrometry) analyses revealed that the contents of the primary constituents (ellagic acid, hyperoside, isoquercitroside, reynoutrin, guaijaverin, auicularin, and quercetin) in RPGLD increased obviously compared with PGLD. These results shed new light on the antidiabetic potential and mechanism of PGL, as well as the “higher efficacy” of the rice-fried processing method in traditional Chinese medicine.

## 1. Introduction

Diabetes mellitus is a metabolic disorder characterized by chronic hyperglycemia resulting from impaired insulin secretion or insulin resistance [1]. More than 400 million people worldwide have suffered diabetes, and this number is expected to increase to 693 million by 2045 [2]. Type 2 diabetes mellitus (T2DM), also called metabolic syndrome, is due to a progressive loss of *β*-cell insulin secretion frequently on the background of insulin resistance and accounts for about 90% of the total number of diabetes patients [3, 4]. Since there is no cure available at present, the treatment and control of T2DM is a challenging task. In clinical, apart from exogenous insulin supplements, different types of oral hypoglycemic drugs such as biguanides, insulin secretagogues, insulin sensitizers, *a*-glucosidase inhibitors, glucagon-like peptide-1 receptor agonists, dipeptidyl peptidase-4 inhibitors, and sodium-glucose cotransporter-2 inhibitors are currently used as the main therapeutic strategy for T2DM [5, 6], whereas the undesirable adverse effects including gastrointestinal discomfort, increasing cardiovascular events, heart failure, and weight gain always accompany with these synthetic drugs [4, 7]. Therefore, nowadays, more and more studies are focusing on the screening of more effective and harmfulless functional ingredients with antidiabetic activity from natural sources.

*P. guajava* Linn. (family: Myrtaceae) is an evergreen shrub native to South America and has been widely cultivated in the tropical and subtropical regions of European, Africa, and Southeast Asia [8]. The dried PGL, with the functions of drying dampness, invigorating the spleen, clearing heat, and detoxifying, have been used for centuries as a folk Chinese medicine to treat diabetes, diarrhea, ulcers, and rheumatism [9, 10]. Modern pharmacology studies have revealed the hypoglycemic efficacy and antidiabetic mechanism of PGL, involving increasing glycogen storage and reducing hormone-sensitive lipase activity, reducing the loss of insulin-positive *β*-cells and insulin secretion, and upregulating the mRNA expressions of IRS-1, phosphatidylinositol 3-kinase, and serine/threonine kinase protein B in livers of diabetic mice [11–13].

As an ancient Chinese pharmaceutic technique, the processing (“paozhi” in Chinese) has been continually explored during the usage of Chinese herbal medicines for higher efficacy, reduced toxicity, and simplified application [14, 15]. The rice-fried processing generally includes removing impurities from raw material, chopping, and then stir-frying with rice until the rice turns yellow. Although both the dried and the rice-fried PGL have been recorded in “Sichuan Province Traditional Chinese Medicine Processing Standards” (2015 edition), whether the rice-fried processing indeed improves the quality of *P. guajava* leaves or not remains considerable controversy. In this study, the HFD/STZ diabetic rat model was constructed to evaluate the in vivo hypoglycemic and hepatoprotective effects of PGLD and RPGLD. The preliminary regulatory mechanism involving the liver glucose metabolism and insulin signal molecule expression was analysed. In the end, a comparative experiment on contents of the primary chemicals and a comprehensive hypoglycemic evaluation by the entropy weight TOPSIS method of PGLD and RPGLD were carried out. Based on the above results, we hope to provide an in-depth understanding of the antidiabetic effect of PGL and a scientific explanation of the advantage of the rice-fried processing in Chinese herbal medicine.

## 2. Materials and Methods

### 2.1. Sample Information

Dried and the rice-fried *P. guajava* (Myrtaceae) leaves were provided by Chengdu Tongling Chinese Medicine Pieces Selection Co. and identified by Professor Yan Zhuyun, Chengdu University of Traditional Chinese Medicine. The rice-fried processing was conducted according to the “Sichuan Province Traditional Chinese Medicine Pieces Processing Standards” (2015 Edition). The decoctions of PGL and RPGL were made as follows: leaves (200 g, dry weight) were soaked in distilled water (1.6 L) for 30 min and then decocted for 30 min; filtered the leaves and decocted them again in distilled water (1.2 L) for 30 min; combined the filtrates and concentrated it to 400 mL, obtaining a final concentration of 0.5 g/mL.

### 2.2. Instruments, Reagents, and Chemicals

PCR amplification instrument (Bio-Rad), ultra-microspectrophotometer (NanoDrop2000), ACCU-CHEK Roche blood glucose meter (Roche Diagnostics), Thermo Varioskan Flash multifunctional microplate reader, UPLC-HRMS equipped with the Q-Exactive quadrupole and electrostatic field orbitrap (Thermo Fisher Scientific), and Agilent 1260 infinity. Acetonitrile (Anhui Tiandi High-Purity Solvent Co.), phosphate and methanol (Chengdu Kelong Chemical Co.), ellagic acid and hyperoside (Chengdu Kloma Biotechnology Co.), isoquercitroside, guaijaverin, and quercetin (Sichuan Weikeqi Biotechnology Co.), reynoutrin and auicularin (Chengdu Aifa Biological Technology Co.), high-fat diet (Chengdu Dashuo Laboratory Animal Co.), streptozotocin (STZ, Sigma company), metformin hydrochloride tablets (China-US Shanghai Squibb Pharmaceutical Co.), HbA1c test kit (Nanjing Jiancheng Institute of Biological Engineering), rat insulin ELISA kit (Yikesai Biotechnology Co.), AxyPrep^TM^ multisource total RNA miniprep kit (Axygen), BeyoFast^TM^ SYBR green qPCR mix (2X) (Biyuntian Biotechnology Company), PrimeScript^TM^ RT reagent kit (TaKaRa company), and primers (Sangon Biotech).

### 2.3. Experimental Animals

Sprague Dawley (SD) male rats (6−8 weeks age, 180−200 g) were obtained from the Chengdu Dashuo Experimental Animal Co. (production license no. SCXK (Sichuan) 2015–030). All rats were housed at 23−25°C in clean, dry plastic cages, and fed with standard pellet diet and water.

### 2.4. Construction and Grouping of the Diabetic Rat Model

The bioassay method was according to our previous study with appropriate modifications [16]. After 7 days of adaptive feeding, SD male rats were fed with a high-fat diet for 28 days, and then after 12 hours of fasting, they were intraperitoneally injected with STZ (45.0 mg/kg). After 3 and 7 days of adaptive feeding, the FBG levels of the rats were measured, among which the rats with a FBG concentration of more than 16.7 mM were considered as the successful diabetic model. Diabetic rats were randomly divided into a model control group (HFD/STZ), a metformin (1.8 mg/kg) positive group (MET), PGLD (2.5 g/kg) treatment group, RPGLD (2.5 g/kg) treatment group, and healthy SD male rats as a normal group (Nor), with 6 rats in each group. All groups were administered intragastrically for 49 days. The animal experiment process was approved by the Experimental Animal Ethics Committee of Chengdu University of Traditional Chinese Medicine (Approval No. 2019–09).

### 2.5. Determination of Bodyweight, FBG, and HbA1c

After administration for 7, 21, 35, and 49 days and then fasting for 12 hours, the bodyweight of rats was measured, and blood was taken from the tail tip to determine the FBG levels. After the last administration, the rats were anesthetized. The blood was taken from the abdominal aorta, and blood cells were separated. The HbA1c levels of the rats were determined according to the HbA1c detection kit.

### 2.6. Histological Examination

After administration, the rats were dissected, and their livers were fixed with 10% formaldehyde, routinely sampled, dehydrated, embedded, and prepared. After H&E staining with basic dye hematoxylin and acid dye eosin, the lesions were observed and described under an optical microscope, and the corresponding lesions of different types in the main description were photographed.

### 2.7. Real-Time qPCR

Total DNA was extracted with AxyPrep^TM^ Multisource Total RNA Miniprep Kit. RNA concentrations and purity were determined with the NanoDrop 2000 ultranucleic acid analyser. RNA integrity was determined with agarose gel electrophoresis. Reverse transcription was performed according to PrimeScript^TM^ RT Reagent Kit instructions, and the rat liver tissue primer sequences are given in Table 1. Real-time qPCR reaction conditions: predenaturation (95°C, 30 s), denaturation (95°C, 5 s), annealing (60°C, 60 s), and fully extended (72°C, 30 s), 40 cycles. The 2^−ΔΔCt^ method was used to measure the relative expression of mRNA.

### 2.8. Determination of the Primary Chemicals in PGLD and RPGLD

#### 2.8.1. Chromatographic and Mass Spectral Conditions

Chromatographic conditions for HPLC quantitative analysis: Zorbax SB-C_18_ column (250 mm × 4.6 mm, 5 *μ*m), mobile phase: acetonitrile (A) and 0.2% phosphoric acid aqueous solution (B), gradient elution (0−15 min, 11%–13.5% A; 15−30 min, 13.5%–18% A; 30−40 min, 18% A; 40−65 min, 18%–49% A; and 65−70 min, 49%–11% A), detection wavelength: 360 nm, injection volume: 10 *μ*L, flow rate: 1.0 mL/min, and column temperature: 35°C. Chromatographic conditions for UPLC-MS/MS analysis: Eclipse Plus C_18_ column (50 mm × 2.1 mm, 1.8 *μ*m), mobile phase: acetonitrile (A) and 0.1% formic acid aqueous solution (B), gradient elution (0−15 min, 11%–13.5% A; 15−30 min, 13.5%–18% A; 30−40 min, 18% A; 40−50 min, 18%–49% A; and 50−60 min, 49%–11% A), detection wavelength: 360 nm, injection volume: 5 *μ*L, flow rate: 0.2 mL/min, and column temperature: 35°C. Mass spectral conditions for UPLC-MS/MS analysis: the apparatus was functioned in positive and negative electrospray ionization modes by an electrospray ionization source. Optimized ESI/MS operational conditions were monitors for instance: spray voltage (3.2 kV), ion source temperature (350°C), sheath gas flow speed (35 arb), auxiliary gas flow speed (10 arb), capillary temperature (320°C), scanning mode (full MS/dd-MS2), scanned range (*m/z*, 100−1500 Da), and gradient impact energy (20, 40, and 60 eV).

#### 2.8.2. Preparation of Test Samples and Reference Substances

The test concentrations of PGLD and RPGLD were both prepared as 0.05 g/mL. The mixed reference substances were ellagic acid, hyperoside, isoquercitroside, reynoutrin, guaijaverin, auicularin, and quercetin, with the initial concentrations (*c*, in methanol) of 171.20, 164.80, 144.32, 166.72, 162.24, 166.72, and 84.84 *μ*g/mL, respectively.

#### 2.8.3. Linear Relationship, Precision, and Stability of the HPLC Method

The analysis method was based on the previous report [17]. Linear relationship test: the above-mixed reference substances were diluted in methanol to obtain a series of mass concentrations (*c*, *c*/2, *c*/4, *c*/8, *c*/16, *c*/32). Inject the references in sequence according to the HPLC chromatographic conditions under “2.7.1.,” record the chromatographic peak area, and draw the standard curves. Then, perform linear regression and 1/*X*^2^ weight and calculate the regression equations and correlation coefficients (*r*). Precision test: prepare the mixed reference substances (*c*/2, 10 *μ*L) for six consecutive injections under the above chromatographic conditions and calculate the RSD of each component peak area. Stability test: analyse the peak areas of the mixed reference substances in initial concentrations (*c*) at 0, 4, 8, 12, 16, and 24 hours and calculate the RSD value of each component. The results showed that the HPLC method had a good linear relationship, precision, and stability.

#### 2.8.4. Sample Recovery Rate and Content Test

Sample recovery rate test: take six PGLD test solutions (0.05 g/mL, each 0.5 mL), add equal volume of mixed reference substances (ellagic acid (47.08 *μ*g/mL), hyperoside (51.50 *μ*g/mL), isoquercitroside (59.53 *μ*g/mL), reynoutrin (18.76 *μ*g/mL), guaijaverin (64.90 *μ*g/mL), auicularin (70.86 *μ*g/mL), and quercetin (4.22 *μ*g/mL)), and calculate the average recovery rate and RSD value of each component. The result showed that the analysis method had a good sample recovery rate. Sample content test: take 6 parallel portions of PGLD and RPGLD (0.05 g/mL) and determine the mass fractions of the seven reference substances under the above chromatographic conditions.

### 2.9. Statistical Analysis

The data were statistically processed using the IBM SPSS Statistics for Windows (version 20, IBM Corp., Armonk, N.Y., USA). Our data were expressed as mean ± standard deviation, and Student's *t*-test or one-way ANOVA was used for the comparison between groups. At the same time, the GraphPad Prism 8 software and the TOPSIS method in SPSSAU were used for comprehensive evaluation.

## 3. Results

### 3.1. Effects of PGLD and RPGLD on Bodyweight of Diabetic Rats

**Figure 1:**
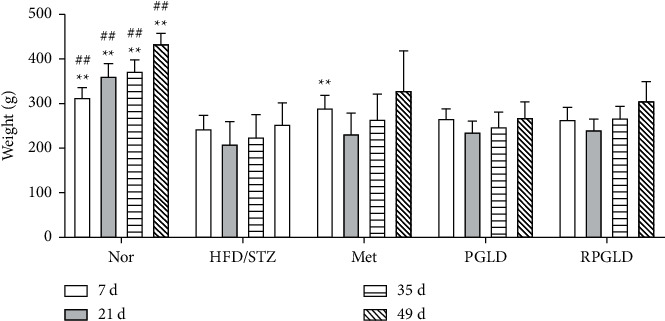
Bodyweight of rat groups (Nor (normal), HFD/STZ (high-fat diet with streptozotocin), Met (metformin), PGLD (*P. guajava* leaves decoctions), and RPGLD (rice-fried *P. guajava* leaves decoctions)) after 7, 21, 35, and 49 days (^*∗*^*P* < 0.05, ^*∗∗*^*P* < 0.01 vs. the HFD/STZ group; ^#^*P* < 0.05, ^##^*P* < 0.01 vs. the PGLD group).

As shown in Figure 1, compared with the Nor group, the bodyweight of the HFD/STZ group significantly decreased at 7, 21, 35, and 49 days. Compared with the HFD/STZ group, the bodyweight in the rat groups of Met, PGLD, and RPGLD exhibited a noticeable increasing trend. After 49 d administration, the RPGLD group showed a little more obvious weight gain than the PGLD group.

### 3.2. Effects of PGLD and RPGLD on FBG of Diabetic Rats

**Figure 2:**
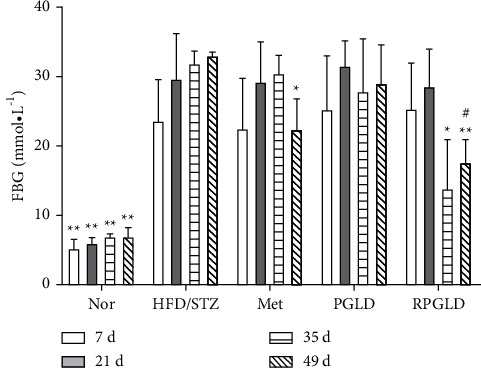
FBG (fasting blood glucose) levels of rat groups (Nor (normal), HFD/STZ (high-fat diet with streptozotocin), Met (metformin), PGLD (*P. guajava* leaves decoctions), RPGLD (rice-fried *P. guajava* leaves decoctions)) after 7, 21, 35, and 49 days (^*∗*^*P* < 0.05, ^*∗∗*^*P* < 0.01 vs. the HFD/STZ group; ^#^*P* < 0.05, ^##^*P* < 0.01 vs. the PGLD group).

As shown in Figure 2, compared with the Nor group, the FBG of the HFD/STZ group significantly increased after 7, 21, 35, and 49 days. Compared with the HFD/STZ group, after 35 d administration, the FBG levels in the rat groups of Met and PGLD showed an obvious decreasing trend, while the RPGLD group declined significantly (*P* < 0.05). After 49 d, the Met and the RPGLD groups both exhibited significantly FBG decreasing activity. In addition, a significant difference occurred between the PGLD and RPGLD groups.

### 3.3. Effects of PGLD and RPGLD on HbA1c of Diabetic Rats

**Figure 3:**
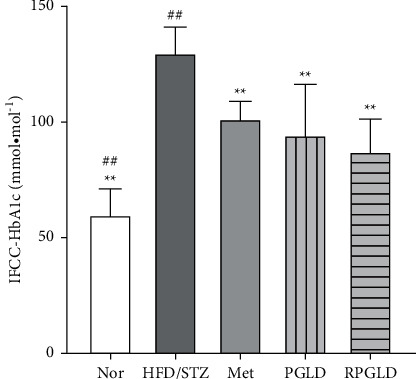
HbA1c (haemoglobin A1c) levels of rat groups (Nor (normal), HFD/STZ (high-fat diet with streptozotocin), Met (metformin), PGLD (*P. guajava* leaves decoctions), and RPGLD (rice-fried *P. guajava* leaves decoctions)) (^*∗*^*P* < 0.05, ^*∗∗*^*P* < 0.01 vs. the HFD/STZ group; ^#^*P* < 0.05, ^##^*P* < 0.01 vs. the PGLD group).

As shown in Figure 3, compared with the Nor group, the HbA1c level of the HFD/STZ group significantly increased. Compared with the HFD/STZ group, the HbA1c levels in the rat groups of Met, PGLD, and RPGLD all significantly declined (*P* < 0.01). The RPGLD group showed a little more obviously downward trend than the PGLD group.

### 3.4. Effects of PGLD and RPGLD on Liver Pathological Changes of Diabetic Rats

**Figure 4:**
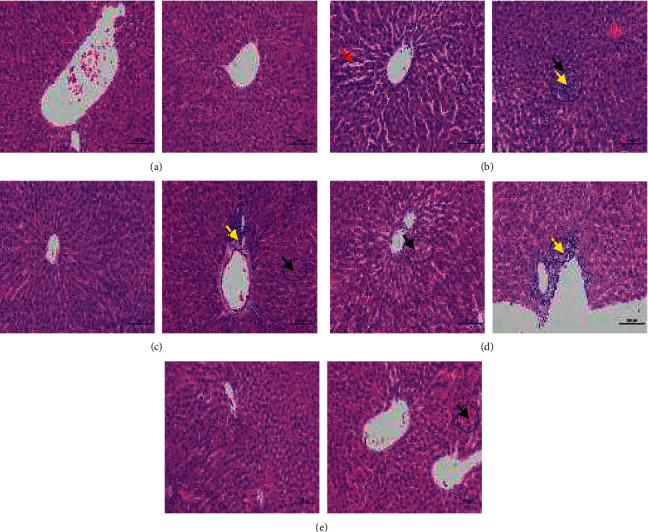
The histological changes of livers in experimental rat groups (a) Nor (normal), (b) HFD/STZ (high-fat diet with streptozotocin), (c) Met (metformin), (d) PGLD (*P. guajava* leaves decoctions), and (e) RPGLD (rice-fried *P. guajava* leaves decoctions). The left and right images of each line refer to different liver regions of the same group. The blue borders mark the observed histological changes in each image.

The results of H&E staining are shown in Figure 4. In the Nor group (a), the liver lobules were clearly structured, the liver cords were neatly arranged, the liver cells were rich in the cytoplasm, and the morphology and structure were normal. In the HFD/STZ group (b), the structure of liver lobules was not complete, while the local necrosis of hepatocytes, the nuclear fragmentation, a small amount of neutrophil infiltration, and the local sinusoidal dilation could be clearly observed. Compared with the HFD/STZ group, the liver lobule structure of the Met group (c) was clear, the liver cords were arranged neatly, the liver cells were rich in the cytoplasm, and the morphology and structure were normal. The PGLD group (d) had a clear liver lobule structure, a neat arrangement of liver cords, and rich cytoplasm. The morphological structure of liver cells was basically normal, and there was a small amount of lymphocyte infiltration around the bile duct in the local portal area. The sinusoids were slightly dilated and squeezed around the central vein. The RPGLD group (e) had clear liver lobules, neatly arranged liver cords, and rich cytoplasm of liver cells. There was no obvious dilation or compression of the liver sinusoids, and only few lymphocyte infiltrations around the bile duct in the portal area could be observed locally. Generally, RPGLD exhibited a better recovery trend on the liver pathological changes of diabetic rats than PGLD.

### 3.5. Effects of PGLD and RPGLD on mRNA Expression of Liver Glucose Metabolism and Insulin Signal Molecules in Diabetic Rats

**Figure 5:**
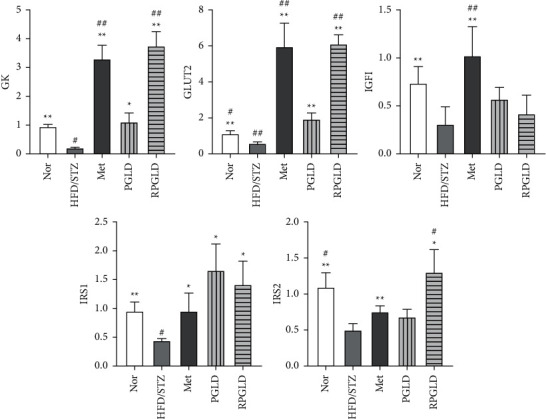
Effects of PGLD (*P. guajava* leaves decoctions) and RPGLD (rice-fried *P. guajava* leaves decoctions) on mRNA expressions ((GK, glucokinase), GLUT2 (glucose transporter 2), IGF-1 (insulin growth factor-1), IRS-1 (insulin receptor substrate-1), and IRS-2 (insulin receptor substrate-2)) of the liver glucose metabolism and insulin signal molecules in diabetic rats (^*∗*^*P* < 0.05, ^*∗∗*^*P* < 0.01 vs. the HFD/STZ group; ^#^*P* < 0.05, ^##^*P* < 0.01 vs. the PGLD group).

As shown in Figure 5, compared with the Nor group, the expression levels of GK, GLUT2, IGF-1, IRS-1, and IRS-2 in the HFD/STZ group decreased significantly. Compared with the HFD/STZ group, the expressions of GK, GLUT2, IGF-1, IRS-1, and IRS-2 in the rat groups of Met, PGLD, and RPGLD all showed an upward ward. Especially, the expressions of GK, GLUT2, and IRS-1 in the PGLD group and the expressions of GK, GLUT2, IRS-1, and IRS-2 in the RPGLD group increased significantly. In addition, compared with the PGLD group, the expressions of GK, GLUT2, and IRS-2 all significantly increased.

### 3.6. Determination and Quantification of the Reference Substances in PGLD and RPGLD

**Figure 6:**
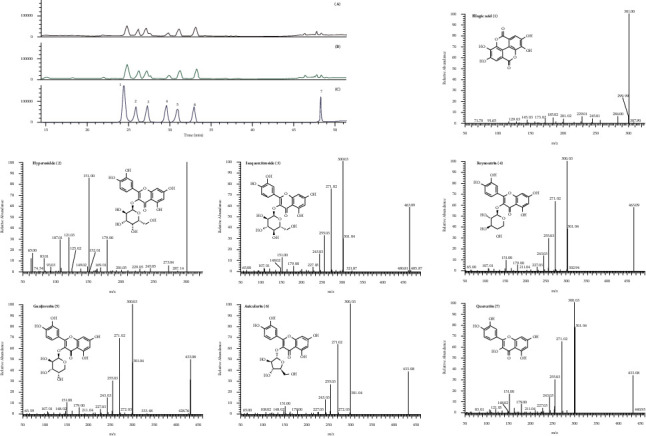
UPLC-MS/MS (ultra-performance liquid chromatography-tandem mass spectrometry) analyses of PGLD (*P. guajava* leaves decoctions) (a), RPGLD (rice-fried *P. guajava* leaves decoctions) (b), and mixed reference substances (c). MS/MS spectra of ellagic acid (**1**), hyperoside (**2**), isoquercitroside (**3**), reynoutrin (**4**), guaijaverin (**5**), auicularin (**6**), and quercetin (**7**).

**Figure 7:**
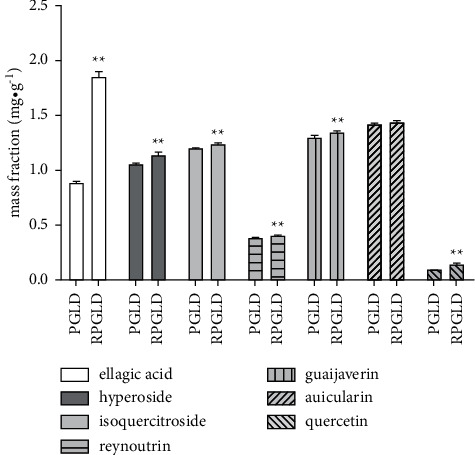
Contents of the reference substances (ellagic acid, hyperoside, isoquercitroside, reynoutrin, guaijaverin, auicularin, and quercetin) in PGLD (*P. guajava* leaves decoctions) and RPGLD (rice-fried *P. guajava* leaves decoctions) (^*∗*^*P* < 0.05 and ^*∗∗*^*P* < 0.01 vs. PGLD).

As shown in Figure 6, the existence of ellagic acid, hyperoside, isoquercitroside, reynoutrin, guaijaverin, auicularin, and quercetin in PGLD and RPGLD was confirmed by the UPLC-MS/MS analyses through comparing with the retention time and MS/MS spectra of the reference substances. The contents of the reference substances in PGLD and RPGLD were quantified by the HPLC analyses, and the linear regression equations are calculated as given in Table 2. Compared with PGLD, the contents of reference substances in RPGLD all significantly increased except for auicularin which only showed a moderate upward trend (Figure 7).

### 3.7. Comprehensive Evaluation

The data of bodyweight, FBG, HbA1c, GK, GLUT2, IGF-1, IRS-1, and IRS-2 were selected for comprehensive analysis, and their weight coefficients were measured as 0.0149, 0.1716, 0.0356, 0.2646, 0.2976, 0.0936, 0.0885, and 0.0336, respectively. The results of entropy TOPSIS were sorted according to relative proximity and obtained as Nor > Met > RPGLD > PGLD > NFD/STZ (Table 3), which indicated the much better antidiabetic effect of RPGLD than that of PGLD.

## 4. Discussion

Previous studies have evaluated the antioxidant, cytoprotective, hypolipidemic, and antidiabetic activities of PGL, and its harboured phytochemicals such as flavonoids, triterpenes, and sesquiterpenes were revealed to possess hypoglycemic and hepatoprotective effects [18]. In this study, the hypoglycemic effect of PGL was also found. After 35 and 49 days of administration, the FBG level of the PGLD group showed an obvious downward trend, and the FBG level of the RPGLD group was significantly lower than the HFD/STZ group. The HbA1c contents in the rat groups of PGLD and RPGLD significantly decreased compared with the HFD/STZ group after 49 d. In addition, noticeable recovery effects of PGLD and the RPGLD on liver pathological changes of diabetic rats were also observed. These results further verified that both PGLD and RPGLD possessed remarkable hypoglycemic and hepatoprotective potential.

The glucose metabolism in the liver via glycogen synthesis and gluconeogenesis plays a key role in maintaining the body's glucose homeostasis. In liver cells, GK could regulate the rates of intrahepatic glucose uptake and glycogen synthesis and inhibit intrahepatic glucose production [19]. GLUT2, promoting the synthesis and secretion of insulin in pancreatic *β*-cells, is responsible for the most important mediator of glucose transport in liver cells [20]. IGF-1 is a polypeptide protein substance structurally similar to insulin, and its insufficient expression or abnormal phosphorylation would lead to insulin resistance [21]. IRS-1 and IRS-2 could reduce blood sugar content by promoting the utilization of glucose and the synthesis of glycogen after phosphorylation [22]. In this study, the upregulated expressions of GK, GLUT2, IGF-1, IRS-1, and IRS-2 mRNAs in the PGLD and RPGLD-treated groups were observed, which suggested that the hypoglycemic effect of PGL might be related to the activation of GK, GLUT2, IGF-1, IRS-1, and IRS-2.

Through comparing the antidiabetic effects of the PGLD and the RPGLD groups, it could be found that after 49 d administration, the RPGLD group exhibited a more significant FBG decreasing effect and an obvious HbA1c declined trend. The recovery effect of RPGLD on the liver pathological changes of diabetic rats was also more noticeable than that of PGLD. The mRNA expressions of GK, GLUT2, and IRS-2 in the RPGLD group all increased significantly compared with the PGLD group. Hence, the above information demonstrated that the rice-fried processing indeed contributed to the improvement of the antidiabetic effect of dried PGL.

Technique for order preference by similarity to ideal solution (TOPSIS) is a commonly used comprehensive evaluation method, which can make full use of the information of the original data, and its results can accurately reflect the gap between the evaluation plans. The TOPSIS's near-ideal solution is the most used approach for multiindex evaluation of the pros and cons of drugs [23]. In this study, the method was used to comprehensively evaluate the antidiabetic effect of PGLD and RPGLD, the results of which showed that on the whole, RPGLD had a much better effect than PGLD. Moreover, the qualitative and quantitative analyses of the seven primary constituents (ellagic acid, hyperoside, isoquercitroside, reynoutrin, guaijaverin, auicularin, and quercetin) in PGLD and RPGLD were carried out, and the results revealed that the contents of the above chemicals in RPGLD were more abundant than those in PGLD. The increased content of primary chemicals in RPGLD was likely attributed to the rice-fried processing, which in turn endowed a better antidiabetic effect in vivo.

## 5. Conclusions

In conclusion, this study demonstrated the antidiabetic effects of RGLD and RPGLD through decreasing FBG and HbA1c levels and alleviating liver pathological changes in diabetic rat models. The related mechanism was at least partially related to the activation of GK, GLUT2, IGF-1, IRS-1 and IRS-2 genes. In general, RPGLD exhibited a better antidiabetic effect than PGLD, which was also verified by the comprehensive evaluation using the entropy weight TOPSIS method. Besides, the higher content of the primary constituents (ellagic acid, hyperoside, isoquercitrin, reynoutrin, guaijaverin, and quercetin) in RPGLD than PGLD was confirmed by HPLC and UPLC-MS/MS analyses. These results provided new insights on the antidiabetic activity and mechanism of *P. guajava* leaves, as well as the “higher efficacy” of the rice-fried processing method.

## Figures and Tables

**Table 1:** Rat liver tissue primer sequences in real-time qPCR.

Primer information	Primer sequence (5′-3′)	Fragment length (bp)
R-GAPDH-S	CTGGAGAAACCTGCCAAGTATG	138
R-GAPDH-A	GGTGGAAGAATGGGAGTTGCT	
R-Gk (rz)-S	GCTTATTTGGAGTTTGACAGGGG	172
R-Gk (rz)-A	CTCAGAAGAACTCCGAACATTGG	
R-Glut2 (rz)-S	GCTCAGCAGTTCTCTGGAATCAA	239
R-Glut2 (rz)-A	TTATCCAGCAACACCAGTCCC	
R-Igf1-S	TGGCACTCTGCTTGCTCACC	180
R-Igf1-A	ACTCATCCACAATGCCCGTCT	
R-Irs1-S	ACACCCGAGACGAACACTTTG	243
R-Irs1-A	AGCCCTTGGGTTTCAGGATAA	
R-Irs2-S	CTCTACCACCACTGTCACCCCA	119
R-Irs2-A	CCAGGATGCTGTTGCCTTCAC	

**Table 2:** Linear regression equations and correlation coefficient of reference substances.

Reference substances	Equations of linear regression	*R* ^2^	Linearity range (*μ*g/mL)
Ellagic acid	*y* = 77.551*x* − 26.235	0.9998	5.35–171.20
Hyperoside	*y* = 106.54*x* − 22.037	0.9998	5.15–164.80
Isoquercitroside	*y* = 132.06*x* − 15.931	0.9998	4.51–144.32
Reynoutrin	*y* = 126.58*x* − 18.39	0.9998	5.21–166.72
Guaijaverin	*y* = 101.08*x* − 2.8876	0.9998	5.07–162.24
Auicularin	*y* = 102.51*x* − 15.844	0.9998	5.21–166.72
Quercetin	*y* = 156.14*x* − 23.523	0.9997	2.64–84.48

**Table 3:** Relative closeness and ranking of index values of each group to the optimal value.

Group	Positive ideal solution distance (D^+^)	Negative ideal solution distance (D^−^)	Relative proximity (C)	Sort results
Nor	5.769	179.898	0.969	1
HFD/STZ	180.02	0.002	0	5
Met	104.719	75.441	0.419	2
PGLD	165.33	14.78	0.082	4
RPGLD	128.416	51.909	0.288	3

## Data Availability

The data used to support the findings of this study are available from the corresponding authors upon request.
